# Hierarchical Multi-Scale Mamba with Tubular Structure-Aware Convolution for Retinal Vessel Segmentation

**DOI:** 10.3390/e27080862

**Published:** 2025-08-14

**Authors:** Tao Wang, Dongyuan Tian, Haonan Zhao, Jiamin Liu, Weijie Wang, Chunpei Li, Guixia Liu

**Affiliations:** 1 College of Computer Science and Technology, Jilin University, Changchun 130012, China; 2 Key Laboratory of Symbolic Computation and Knowledge Engineering of Ministry of Education, Jilin University, Changchun 130012, China; 3 Department of Information Engineering and Computer Science, University of Trento, 38123 Trento, Italy; 4 School of Science, Jimei University, Xiamen 361021, China; 5 School of Computer Science and Engineering, Guangxi Normal University, Guilin 541001, China

**Keywords:** retinal vessel segmentation, tubular structure-aware convolution, Mamba, multi-scale fusion, attention mechanism

## Abstract

Retinal vessel segmentation plays a crucial role in diagnosing various retinal and cardiovascular diseases and serves as a foundation for computer-aided diagnostic systems. Blood vessels in color retinal fundus images, captured using fundus cameras, are often affected by illumination variations and noise, making it difficult to preserve vascular integrity and posing a significant challenge for vessel segmentation. In this paper, we propose HM-Mamba, a novel hierarchical multi-scale Mamba-based architecture that incorporates tubular structure-aware convolution to extract both local and global vascular features for retinal vessel segmentation. First, we introduce a tubular structure-aware convolution to reinforce vessel continuity and integrity. Building on this, we design a multi-scale fusion module that aggregates features across varying receptive fields, enhancing the model’s robustness in representing both primary trunks and fine branches. Second, we integrate multi-branch Fourier transform with the dynamic state modeling capability of Mamba to capture both long-range dependencies and multi-frequency information. This design enables robust feature representation and adaptive fusion, thereby enhancing the network’s ability to model complex spatial patterns. Furthermore, we propose a hierarchical multi-scale interactive Mamba block that integrates multi-level encoder features through gated Mamba-based global context modeling and residual connections, enabling effective multi-scale semantic fusion and reducing detail loss during downsampling. Extensive evaluations on five widely used benchmark datasets—DRIVE, CHASE_DB1, STARE, IOSTAR, and LES-AV—demonstrate the superior performance of HM-Mamba, yielding Dice coefficients of 0.8327, 0.8197, 0.8239, 0.8307, and 0.8426, respectively.

## 1. Introduction

In recent years, visual impairments caused by ocular diseases have risen significantly, affecting more than 2.2 billion people worldwide [1]. Consequently, early screening and timely intervention for retinal diseases are crucial. Analyzing the retinal vascular system allows for the assessment of vascular morphology, which is essential for diagnosing retinal lesions and cardiovascular-related diseases [2]. Retinal vasculitis, an inflammatory condition, leads to significant changes in the retinal vasculature [3], while age-related macular degeneration often involves vascular abnormalities, particularly affecting the central region of the retina [4]. Diabetic retinopathy, a common complication of diabetes, manifests as microvascular changes detectable through retinal vascular observation [5], while hypertension induces arteriosclerosis and hemorrhages in the retinal vasculature [6]. Retinal vessel segmentation enables the extraction of morphological information, such as vessel diameter, thickness, curvature, color, and contrast. However, segmentation tasks face numerous challenges, including poor image quality, vascular complexity, and various interfering factors. In the past, ophthalmologists manually performed retinal vessel segmentation, which was time-consuming, labor-intensive, and costly, making it difficult to meet the demands of large-scale screening [2]. To address these issues, researchers have increasingly turned to artificial intelligence-assisted retinal disease screening, leading to the development of automated retinal vessel segmentation methods.

The rapid advancement of artificial intelligence, particularly in deep learning and image processing, has significantly advanced research on retinal vessel segmentation. From the perspective of visual representation, the development of deep learning-based computer vision models can be categorized into two main trajectories: Convolutional Neural Networks (CNNs) and Vision Transformers (ViTs). CNNs, characterized by local connectivity, weight sharing, and translational invariance, naturally align with the inductive bias required for modeling image information, making them particularly well-suited for early visual representation tasks. In retinal vessel segmentation, CNN-based models have played a pivotal role, including Weighted Res-UNet [7], Vessel-Net [8], RVGSeg-Net [9], FANet [10], Genetic U-Net [11], OCE-Net [12], and BINet [13]. Despite the strong performance of CNNs in image segmentation tasks, their receptive field is inherently limited by the convolutional kernel size, making it difficult to capture long-range dependencies. Moreover, CNNs primarily focus on local feature modeling, which restricts their effectiveness in capturing fine-grained structures and complex contextual relationships. ViTs, on the other hand, leverage the self-attention mechanism to dynamically adjust their focus on different features of the input data. By partitioning images into small patches and converting them into vectors, ViTs generate high-level feature representations through multiple layers of self-attention and feedforward networks, effectively capturing long-range dependencies and complex contextual relationships. Since their inception, ViTs have demonstrated the ability to advance a wide range of visual tasks, including image classification, object detection, semantic segmentation, image restoration, and video understanding [14]. In this context, retinal vessel segmentation studies based on ViT architectures have emerged rapidly, showcasing their potential in medical image analysis, including DA-Net [15], MTPA_Unet [16], GT-DLA-dsHFF [17], SGAT-Net [18], G2ViT [19], and TCU-Net [20]. Although Transformers excel at modeling long-range dependencies and global context, they present certain limitations in image segmentation tasks. Specifically, they often require large-scale annotated datasets for effective training, which makes them prone to overfitting in small-sample domains such as medical imaging. Additionally, the lack of inherent inductive biases constrains their ability to capture fine-grained boundaries and small structures.

Recently, the Mamba model has garnered widespread attention from researchers and has achieved significant results in time-series analysis, text classification, and image segmentation [21]. Particularly in medical image segmentation, the Mamba model has provided technical support for the precise segmentation of complex lesion areas, owing to its efficient handling of long-range dependencies and accurate feature extraction capabilities [22]. This has enabled Mamba to excel in specialized medical tasks such as cell nucleus segmentation (LoG-VMamba [23]) and retinal vessel segmentation (e.g., Serp-Mamba [24], OCTAMamba [25]), significantly improving the accuracy and efficiency of automated diagnosis and establishing it as a key tool in the field of intelligent healthcare. Nevertheless, Mamba-based methods still face several limitations when applied to retinal vessel segmentation. First, as an emerging architecture, Mamba remains in an exploratory stage in the domain of spatial feature modeling for fine-grained biomedical structures, with no mature or standardized paradigms established to date. Its sequence-centric processing may inadvertently suppress spatial locality, leading to suboptimal delineation of narrow vessels and bifurcation points. Second, current Mamba models are prone to significant computational overhead, which may limit their applicability in clinical scenarios that require lightweight and real-time inference.

To address these issues, we propose HM-Mamba, a U-shaped network that integrates Mamba and CNN architectures for retinal vessel segmentation. HM-Mamba is built upon the classic U-Net architecture and incorporates several key designs tailored to the unique characteristics of color fundus images. Specifically, the proposed network integrates tubular-structure-aware convolutional modules to better capture the elongated and continuous nature of retinal vessels. In addition, a frequency-domain enhanced Mamba block is introduced to improve global feature representation, while multi-scale fusion and attention mechanisms are employed to strengthen contextual understanding and feature refinement across different resolutions. These architectural innovations are carefully designed to address the challenges posed by the fine-grained and topologically complex vascular patterns in retinal images. Extensive experiments were conducted to evaluate the effectiveness of the proposed model and to identify optimal hyperparameter settings across various benchmarks. Our main contributions are as follows:

- We design a tubular structure-aware convolution and a multi-scale fusion module, which jointly enhance vessel continuity and integrity while improving the representation of both major trunks and fine branches across varying receptive fields.
- We introduce a Fourier-transform-based multi-branch Mamba fusion module to effectively model long-range dependencies across both high-frequency and low-frequency components, thereby enhancing the network’s capability to capture complex spatial patterns.
- We propose a Hierarchical Multi-scale Interactive Mamba Block that hierarchically integrates multi-level encoder features through gated Mamba-based global context modeling and residual connections, enabling effective multi-scale semantic fusion and mitigating detail loss during downsampling.

## 2. Related Work

### 2.1. Retinal Vessel Segmentation

Deep learning techniques have provided powerful tools for retinal vessel segmentation, continuously advancing both research and clinical applications in this field. Various methods based on network architectures such as Convolutional Neural Networks (CNNs), Transformers, and Mamba have emerged rapidly, offering innovative solutions to address the challenges in retinal vessel segmentation [2,26]. These methods automatically extract image features, handle complex vascular structures, and overcome the limitations of traditional approaches in terms of accuracy, efficiency, and robustness, significantly improving segmentation performance. Zhang et al. [27] proposed a method that integrates a pyramid channel attention module into the U-Net architecture and replaces standard convolutional blocks with pre-activated residual convolution blocks, enhancing the extraction of small vessels in retinal images to improve performance and generalization. Zhao et al. [28] introduced a multi-modal feature co-learning framework, enabling 2D and 3D models to collaborate and learn from each other, effectively leveraging the convenience of 2D projection maps and the structural depth of 3D volumes to improve retinal vessel segmentation. Tong et al. [29] proposed LiViT-Net, a lightweight Transformer-based model for retinal vessel segmentation that combines MobileViT+ with novel local representations and a joint loss function, effectively addressing the challenges of scale variation and foreground–background imbalance. Liu et al. [30] introduced SpecFormer, which utilizes sparse spectral neural operators and dual-attention blocks to capture low-frequency components in the Fourier domain, effectively segmenting complex vascular-like structures by capturing long-range dependencies and contextual information.

### 2.2. Mamba for Segmentation

Mamba is an emerging selective state space model initially applied in natural language processing, which effectively captures long-range dependencies while significantly reducing computational overhead. Compared to CNNs, Mamba overcomes the limitations of their local perceptual capacity and, when compared to Transformers, improves computational efficiency by avoiding quadratic complexity [31]. Following the successful resolution of conversion challenges between Mamba’s application in graph processing and visual tasks, as seen in methods like Graph-Mamba [32] and VMamba [33], researchers have continued to explore Mamba’s potential in various vertical-domain tasks. Currently, Mamba-based approaches are rapidly gaining momentum in the medical imaging field, with numerous innovative methods emerging. These studies cover a range of areas, including image segmentation, disease detection, and image registration, highlighting Mamba’s unique advantages and vast potential in handling medical images. In terms of network architecture, Mamba enhances both local feature extraction and long-range dependency modeling by integrating with CNNs or Transformers, with representative models such as U-Mamba [34], Mamba-UNet [22], SwinUMamba [35], T-Mamba [36], and VM-UNet [37]. Additionally, lightweight and efficient design strategies, such as model compression, knowledge distillation, and deformable convolutions, have been adopted to optimize computational complexity. Overall, Mamba and its variants have driven innovations and advancements in medical image segmentation, improving accuracy, computational efficiency, and adaptability to complex tasks while maintaining high performance. Notable models include LightM-UNet [38], UltraLight VM-UNet [39], and nnMamba [40]. In summary, Mamba and its variants continue to propel innovation and progress in medical image segmentation, excelling in accuracy, computational efficiency, and their ability to tackle complex tasks.

### 2.3. Multi-Scale Feature Fusion

Multi-scale features refer to complementary hierarchical representations constructed via multi-resolution sampling and analysis of input signals (e.g., images, videos, and time-series data), where finer granularities capture localized details while coarser granularities encode global contextual patterns [41,42]. Multi-scale feature fusion has garnered increasing attention in the medical image segmentation field, unlocking novel possibilities for refined solutions that significantly enhance recognition accuracy for complex pathological structures and subtle anatomical tissues, particularly in addressing intricate boundary delineation and scale-variant target characterization [43,44]. In the field of medical image segmentation, the pyramid architecture enhances global–local semantic coupling through multi-resolution feature stacking; the multi-branch parallel network uses heterogeneous convolution kernels or resolution branches to achieve cross-scale feature complementarity; the cross-scale fusion strategy optimizes the collaborative expression of fine-grained boundaries and coarse-grained morphology through dynamic weight allocation; and the scale-aware adaptive mechanism gives the model robustness to target size distribution [45]. These technologies significantly improve the accuracy of tumor heterogeneity region characterization and low-contrast tissue interface segmentation through hierarchical feature decoupling and adaptive context aggregation, effectively improving the performance of medical image segmentation tasks. For example, Yang et al. [46] proposed MSFFU-Net, a novel U-Net-based framework incorporating Inception-based architectural modules into the encoder to achieve hierarchically enhanced multi-scale feature representation. Zhou et al. [47] replaced the original convolutional layer with an improved dilated residual module that combined a dual attention mechanism with different dilation rates to extract multi-scale vascular features and achieve high-precision retinal vascular segmentation.

## 3. Methods

### 3.1. Network Structure

**Figure 1:**
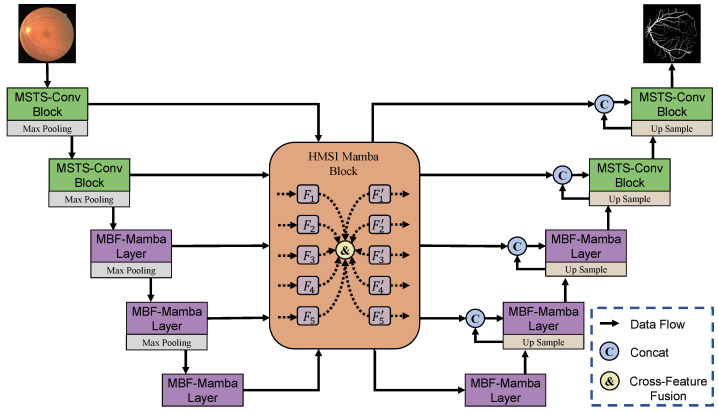
Overall architecture of the HM-Mamba model. MSTS-Conv Block: Multi-Scale Tubular Structure-Aware Convolution Block; MBF-Mamba Layer: Multi-Branch Fourier-Mamba Layer; HMSI-Mamba Block: Hierarchical Multi-Scale Interactive Mamba Block.

Figure 1 illustrates the overall framework of the proposed HM-Mamba, a two-phase U-shaped architecture specifically designed for retinal vessel segmentation, consisting of a Convolution Phase and a Multi-Branch Vision Mamba Layer Phase. Given an input image, the encoder first extracts shallow features using a convolutional block composed of a standard convolutional layer and a multi-scale, vessel-structure-aware convolution module, aiming to enhance and refine the feature representations. For deeper feature extraction, the Mamba block is employed, integrating state-space modeling with Fourier filtering to effectively capture multi-scale features and model long-range dependencies. Symmetrically, the decoder consists of three Mamba blocks followed by two convolutional blocks.

### 3.2. MSTS-Conv Block

**Figure 2:**
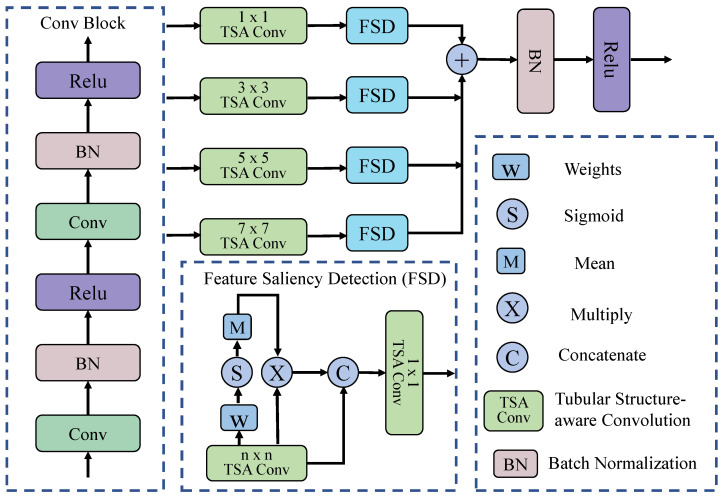
Overview of the Multi-Scale Tubular Structure-Aware Convolution Block. The module first extracts features using basic convolution blocks, then applies tubular structure-aware convolution and feature saliency detection at multiple scales, and finally fuses, normalizes, and activates the features to enhance the modeling capability of tubular structures.

**Tubular Structure-Aware Convolution:** As shown in Figure 2, given an input feature map X$\in R^{B\times C\times H\times W}$, the convolution proceeds through the following steps. We first establish a normalized coordinate grid $G\in R^{k\times k\times 2}$ for convolutional kernels of size k×k, where each position defines centered spatial offsets relative to the kernel’s central pixel:

(1) $$G\left(i,j\right)={\left[i-\frac{k-1}{2},\;j-\frac{k-1}{2}\right]}^{T},\;\forall i,j\in \left\{0,1,\ldots ,k-1\right\}$$

To parameterize orientation selectivity, each channel c∈{1,…,C} learns an angular projection $\theta_{c}\in \left[0,\pi \right)$ via a differentiable transformation, which can be expressed as:

(2) $$\theta_{c}=\pi \cdot \sigma \left(\alpha_{c}\right),\;\sigma \left(z\right)=\frac{1}{1+e^{-z}}$$

where $\alpha_{c}\in R$ is a learnable parameter. Next, directional sensitivity is encoded through harmonic modulation to generate anisotropic filtering kernels, thereby enhancing alignment with vascular structures. This can be computed as follows:

(3) $$D_{c}\left(i,j\right)=\cos\left(2\pi \left(G\left(i,j,0\right)\cos\theta_{c}+G\left(i,j,1\right)\sin\theta_{c}\right)\right)$$

These orientation-selective components are combined with learnable Gaussian bases $G_{c}\in R^{k\times k}$, initialized as:

(4) $$G_{c}^{\left(0\right)}\left(i,j\right)=\exp\left(-\frac{{∥G\left(i,j\right)∥}_{2}^{2}}{2\tau^{2}}\right)$$

where τ controls the initial spatial bandwidth. The kernel synthesis process combines these elements through normalized Hadamard products, which can be formulated as:

(5) $$K_{c}^{\prime }\left(i,j\right)=\frac{G_{c}\left(i,j\right)⊙D_{c}\left(i,j\right)}{\sqrt{\sum_{i^{\prime },j^{\prime }}{\left(G_{c}\left(i^{\prime },j^{\prime }\right)⊙D_{c}\left(i^{\prime },j^{\prime }\right)\right)}^{2}+\epsilon }}$$

where ⊙ denotes the element-wise (Hadamard) product between corresponding entries of the two matrices, and $\epsilon =10^{-6}$ to ensure numerical stability. Finally, a depthwise convolution is applied using these orientation-specialized kernels. The computation is given as follows:

(6) $$Y_{c}\left(n,h,w\right)=\sum_{i=0}^{k-1}\sum_{j=0}^{k-1}X_{c}\left(n,h+i-p,w+j-p\right)\cdot K_{c}^{\prime }\left(i,j\right)$$

where p=⌊k/2⌋ maintains spatial resolution, and for all n∈{1,…,B}, h∈{1,…,H}, and w∈{1,…,W}. Finally, the complete operation is expressed as Y, implementing parameter-efficient, channel-specific filtering while preserving structural homogeneity and adapting to local vascular geometry through differentiable orientation learning. Here, Y is formulated as in Equation (7):

(7) $$Y=\overset{C}{\underset{c=1}{⨁}}\left(X_{c}*K_{c}^{\prime }\right)$$

where ∗ denotes 2D convolution and ⨁ represents channel-wise concatenation. Finally, we define this complete operation as a Tubular Structure-aware Convolution (TSA-Conv), denoted by the following operator:

(8) $$X_{o}=TSAConv\left(X\right)$$

This design maintains architectural simplicity while dynamically adapting to tubular anatomical structures such as retinal vessels and elongated lesions through differentiable harmonic filtering.

**Multi-Scale Fusion**: To effectively enhance vessel tubular features, we introduce a dual-stage multi-scale feature fusion module that incorporates two standard convolutional blocks for enhanced feature representation, along with a dedicated multi-scale fusion mechanism designed to adaptively refine tubular structures. Let $X_{in}\in R^{C\times H\times W}$ denote the input feature map. In the first phase, hierarchical features $F_{\mathrm{base}}$ are extracted through two sequential convolutions:

(9) $$F_{1}=Relu\left(BN(W_{1}*X_{in})\right)$$

(10) $$F_{\mathrm{base}}=Relu\left(BN(W_{2}*F_{1})\right)$$

where $W_{1},W_{2}\in R^{3\times 3}$ are convolutional kernel weights, BN denotes batch normalization, and dropout regularization is applied to mitigate overfitting.

In the second stage, multi-scale tubular structure-aware modules are incorporated to model vascular continuity and capture morphological features across scales, effectively suppressing false segmentations in non-tubular regions and enhancing the understanding of vascular topology. We perform the TSA-Conv operation on the input feature $F_{\mathrm{base}}$ using convolution kernels of size 1×1, 3×3, 5×5, and 7×7, respectively. Let this operation be denoted as ${\mathrm{TSAConv}}_{k}\left(\cdot \right)$, where *k* represents the convolution kernel size. The execution of multi-scale feature extraction is as follows:

(11) $$F_{k}=TSAConv_{k}\left(F_{\mathrm{base}}\right),\;k\in \left\{1,3,5,7\right\}$$

Each feature $F_{k}$ generated by TSAConv is then fed into the Feature Saliency Detection (FSD) module for saliency-oriented feature modeling. Let the FSD module be the function FSD(·), which can be expressed as follows:

(12) $${\hat{F}}_{k}=FSD\left(F_{k}\right)$$

Finally, the outputs of the FSD modules across all scales are summed to obtain the fused feature map. Specifically, the saliency-enhanced features ${\hat{F}}_{k}$ are summed, followed by Batch Normalization (BN) and ReLU activation to produce the final output:

(13) $$F_{\mathrm{fused}}=Relu\left(BN\left(\sum_{k\in \{1,3,5,7\}}{\hat{F}}_{k}\right)\right)$$

The FSD module integrates both channel-wise and spatial saliency information in a lightweight yet effective manner. Given an input feature map $F_{k}$, the module applies a learnable 1×1 convolution to generate a raw per-pixel importance map $W_{c}$, as shown in Equation (13):

(14) $$W_{c}=Conv_{1\times 1}\left(F_{k}\right),\;W_{c}\in R^{C\times H\times W}$$

This operation enables the model to learn a task-specific weighting for each spatial location in every channel. Next, the sigmoid function is applied element-wise to convert the raw scores $W_{c}$ into probabilistic attention weights. It follows that:

(15) $$P_{c}=\sigma \left(W_{c}\right)$$

Here, $P_{c}\in {\left[0,1\right]}^{C\times H\times W}$ represents the probability of importance for each spatial location in every channel. Then, a global attention coefficient for each channel is computed by averaging over the spatial dimensions:

(16) $$\alpha =\frac{1}{H\cdot W}\sum_{h=1}^{H}\sum_{w=1}^{W}P_{c}\left(:,h,w\right),\;\alpha \in R^{C\times 1\times 1}$$

Finally, channel-wise recalibration is performed by reweighting the input feature map using the derived attention coefficients:

(17) $${\hat{F^{\prime }}}_{k}=F_{k}⊗\alpha$$

where ⊗ denotes element-wise multiplication with broadcasting across spatial dimensions.

To further refine spatial regions of interest, the channel-attended feature ${\hat{F^{\prime }}}_{k}$ is concatenated with the original feature map $F_{k}$, and TSAConv is applied to reduce the channel dimension to match that of the base feature map $F_{\mathrm{base}}$. Consequently, we derive the following:

(18) $$F_{concat}=TSAConv\left(Concat\left({\hat{F^{\prime }}}_{k},F_{k}\right)\right)$$

### 3.3. Multi-Branch Fourier-Mamba Block

**Figure 3:**
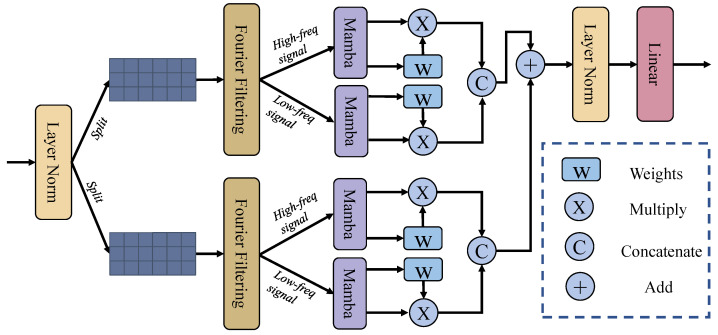
Overview of the Multi-Branch Fourier-Mamba Block. This module first normalizes the input features and then partitions them into feature blocks. It then performs Fourier-based high- and low-frequency decomposition, followed by Mamba processing, respectively.

As depicted in Figure 3, the Multi-Branch Fourier-Mamba Block primarily comprises two components: the Fourier transform and the Mamba layer. The core idea is to apply Fourier transforms to extract complementary high- and low-frequency components, followed by employing Mamba modules to learn frequency-aware global contextual representations. Take an input feature $X_{s}\in R^{B\times C\times H\times W}$ from stage *s*, where s∈3,4,5. First, the input is normalized, and the features are decoupled:

(19) $$X_{\mathrm{flat}}=Reshape\left(X_{s}\right)\in R^{B\times N\times C},\;N=H\times W$$

(20) $$X_{\mathrm{norm}}=\mathrm{LN}\left(X_{\mathrm{flat}}\right)$$

(21) $$X_{1},X_{2}=Split\left(X_{\mathrm{norm}},2\right),X_{i}\in R^{B\times N\times C/2}$$

where LN denotes LayerNorm.

Then, frequency-domain filtering is performed separately on the two branches, where each input undergoes a Fourier transform, frequency component decomposition, and inverse transformation. For the two input feature maps $X_{1}$ and $X_{2}$, frequency domain filtering is independently applied to each branch. Specifically, both inputs are first transformed into the frequency domain using the Fourier transform:

(22) $${\hat{X}}_{1}=F\left(X_{1}\right),\;{\hat{X}}_{2}=F\left(X_{2}\right)$$

Low-frequency and high-frequency components are then extracted using the operator M(·,·), with thresholds μ and ν, respectively. The corresponding components are subsequently transformed back to the spatial domain via the inverse Fourier transform:

(23) $${\hat{X}}_{1,low}=M\left(X_{1},\mu \right),\;X_{1,low}=F^{-1}\left({\hat{X}}_{1,low}\right)$$

(24) $${\hat{X}}_{2,low}=M\left(X_{2},\mu \right),\;X_{2,low}=F^{-1}\left({\hat{X}}_{2,low}\right)$$

(25) $${\hat{X}}_{1,high}=M\left(X_{1},\nu \right),\;X_{1,high}=F^{-1}\left({\hat{X}}_{1,high}\right)$$

(26) $${\hat{X}}_{2,high}=M\left(X_{2},\nu \right),\;X_{2,high}=F^{-1}\left({\hat{X}}_{2,high}\right)$$

where M denotes the high-frequency and low-frequency information extraction operations, with μ and ν as the corresponding thresholds for high-frequency and low-frequency filtering, respectively, and F and $F^{-1}$ representing the Fourier transform and its inverse. As a result, each input produces two spatial-domain outputs corresponding to its low- and high-frequency components.

Next, for the frequency domain feature maps $X_{1,\mathrm{low}}$, $X_{1,\mathrm{high}}$, $X_{2,\mathrm{low}}$, $X_{2,\mathrm{high}}$ obtained in the previous step, first, the representation features are extracted through the Mamba operation:

(27) $$Y_{ij}=\mathrm{Mamba}\left(X_{ij}\right)\;\forall i\in \left\{1,2\right\},j\in \left\{\mathrm{low},\mathrm{high}\right\}$$

Here, Mamba is used to capture the long-range dependencies in each feature map. The process can be simply expressed as:

(28) $$h_{t}={\bar{A}}_{t}h_{t-1}+{\bar{B}}_{t}x_{t}$$

(29) $$y_{t}=C_{t}h_{t}+D_{t}x_{t}$$

where ${\bar{A}}_{t}=e^{\Delta_{t}A}$, ${\bar{B}}_{t}={\left(\Delta_{t}A\right)}^{-1}\left(e^{\Delta_{t}A}-I\right)\Delta_{t}B$ and the parameters A,B,C,D, and Δ are dynamically generated via a selective scanning mechanism. At the same time, for the input feature $X_{i,j}$, the weight tensor is first constructed through a learnable linear projection and then normalized to the unit interval using a sigmoid activation function to obtain the normalized weight $W_{i,j}$. This mechanism realizes adaptive feature importance calibration and interference signal suppression in the channel dimension by dynamically evaluating the significance distribution of multi-frequency features. Next, the previously obtained $Y_{i,j}$ is multiplied with its corresponding $W_{i,j}$ to achieve feature-level attention weighting, strengthen important feature responses, and suppress noise. This process can be expressed by the following formula:

(30) $${\hat{Y}}_{ij}=Y_{ij}⊙w_{ij}$$

Here, ⊙ represents the Hadamard product. Next, the concatenation operation along the channel dimension retains the complementary information of multi-resolution features and forms a hierarchical feature representation as shown below:

(31) $$Z_{1}=\mathrm{Concat}\left({\hat{Y}}_{1,\mathrm{low}},{\hat{Y}}_{1,\mathrm{high}}\right)$$

(32) $$Z_{2}=\mathrm{Concat}\left({\hat{Y}}_{2,\mathrm{low}},{\hat{Y}}_{2,\mathrm{high}}\right)$$

Finally, the features from the two branches are fused, followed by layer normalization to reduce internal covariate shift and stabilize the training process:

(33) $$Z=LN(Z_{1}+Z_{2})$$

(34) $$Z’=Reshape\left(Z^{⊤}\right)\in R^{B\times D\times H\times W}$$

### 3.4. Hierarchical Multi-Scale Interactive Mamba Block

**Figure 4:**
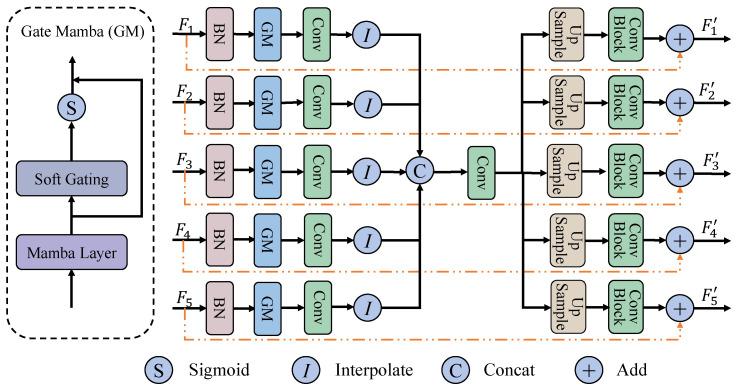
Overview of the Hierarchical Multi-Scale Interactive Mamba Block. This module fuses features from different encoding stages through a series of operations, then adds them to the input features to obtain enhanced representations.

As shown in Figure 4, unlike the skip connections in traditional U-shaped networks, which directly concatenate the features of the encoder and decoder, we introduce the Hierarchical Multi-Scale Interactive Mamba Block (HMSI-Mamba Block) within the skip connections to more effectively exploit multi-scale information. Suppose the input consists of five-stage encoder features ${\left\{F_{i}\in R^{B\times C_{i}\times H_{i}\times W_{i}}\right\}}_{i=1}^{5}$, where C=[64,128,256,512,1024] denotes the predefined number of channels. Initially, features from different encoder stages are subjected to batch normalization to mitigate inter-stage distribution discrepancies. Subsequently, a Gated Mamba (GM) module is employed to capture global contextual representations through the Mamba layer. A learnable channel-wise attention mechanism is then applied to adaptively modulate the response strength of each feature channel, thereby enhancing the overall discriminative capacity of the representations. The overall process can be formulated as $F_{i}^{*}=\mathrm{GM}\left(F_{i}\right)$ and can be further decomposed into the following steps:

(35) $$X^{*}=Mamba(BN\left(Reshape\left(X\right)\right)$$

(36) $$X_{gm}^{*}=Reshape^{-1}\left(X^{*}\right)\cdot \sigma \left(\gamma \right)$$

where X denotes the input feature map, σ(·) represents the Sigmoid activation function, and γ is a learnable parameter associated with the channel dimension.

Subsequently, the previously obtained features $F_{i}^{*}$ are first processed through a 1×1 convolution to align their channel dimensions, producing ${\tilde{F}}_{i}$. These features are then upsampled to a unified spatial resolution of $H_{s}\times W_{s}$, yielding ${\hat{F}}_{i}$. Finally, all spatially aligned features ${\hat{F}}_{i}$ are concatenated along the channel dimension to construct the final fused representation, denoted as $F_{\mathrm{fused}}$. This process can be expressed as:

(37) $${\hat{F}}_{i}={\mathrm{Conv}}_{1\times 1}\left(F_{i}^{*}\right)$$

(38) $${\hat{F}}_{i}=I\left({\tilde{F}}_{i},\mathrm{size}=\left(H_{s},W_{s}\right)\right)$$

(39) $$F_{\mathrm{fused}}=\mathrm{Concat}\left({\hat{F}}_{1},{\hat{F}}_{2},{\hat{F}}_{3},{\hat{F}}_{4},{\hat{F}}_{5}\right)$$

where *I* denotes the interpolation operation. Similarly, a 1×1 convolution is applied to the fused feature map to project it onto a predefined channel dimension, yielding the refined representation $F_{\mathrm{fused}}^{*}$. At this stage, the features extracted from different encoder levels are effectively integrated, resulting in a unified representation enriched with multi-scale semantic information. Subsequently, $F_{\mathrm{fused}}^{*}$ is individually resampled to match the spatial resolution of each input feature map ${\left\{F_{i}\in R^{B\times C_{i}\times H_{i}\times W_{i}}\right\}}_{i=1}^{5}$. To further improve the representational capacity of the features, two standard convolutional blocks are employed to enhance the model’s ability to capture and integrate semantic information across multiple scales. This mapping is formally defined as:

(40) $${\tilde{F}}_{i}=C\left(\mathrm{Resize}(F_{\mathrm{fused}}^{*},H_{i},W_{i})\right),\;i=1,2,3,4,5$$

where Resize(·) denotes an upsampling or downsampling operation, and C(·) denotes a standard convolution operation consisting of two Conv-BN-ReLU layers. Finally, to enhance the original features with enriched semantic cues, a residual connection is introduced between each ${\tilde{F}}_{i}$ and its corresponding input feature $F_{i}$, formulated as follows:

(41) $$F_{i}^{\prime }=F_{i}+{\tilde{F}}_{i},\;i\in \left\{1,2,3,4,5\right\}$$

### 3.5. Loss Function

To effectively train the proposed HM-Mamba model, we adopt the hybrid BceDice loss introduced in VM-UNet [37]. This composite loss function synergistically combines the advantages of binary cross-entropy (BCE) and Dice loss, aiming to optimize both pixel-level classification accuracy and global region-level overlap. Concretely, given the predicted segmentation probability map $\hat{Y}$ and the ground truth mask *Y*, the loss is defined as:

(42) $$L_{\mathrm{BceDice}}=\lambda_{\mathrm{bce}}\cdot L_{\mathrm{BCE}}\left(\hat{Y},Y\right)+\lambda_{\mathrm{dice}}\cdot L_{\mathrm{Dice}}\left(\hat{Y},Y\right)$$

where $L_{\mathrm{BCE}}$ is the binary cross-entropy loss:

(43) $$L_{\mathrm{BCE}}=-\frac{1}{N}\sum_{i=1}^{N}\left[y_{i}\log{\hat{y}}_{i}+\left(1-y_{i}\right)\log\left(1-{\hat{y}}_{i}\right)\right]$$

and $L_{Dice}$ is the soft Dice loss, formulated as follows:

(44) $$L_{\mathrm{Dice}}=1-\frac{2\sum_{i=1}^{N}y_{i}{\hat{y}}_{i}+\epsilon }{\sum_{i=1}^{N}y_{i}+\sum_{i=1}^{N}{\hat{y}}_{i}+\epsilon }$$

From an information-theoretic perspective, the BCE component seeks to minimize the conditional entropy $H(Y|\hat{Y})$, thereby reducing predictive uncertainty, while the Dice term implicitly maximizes the mutual information between $\hat{Y}$ and *Y*, promoting strong region-level consistency and overlap. This joint formulation is especially beneficial in medical and natural image segmentation tasks where foreground–background imbalance and structural coherence are critical. Accordingly, we set $\lambda_{\mathrm{bce}}=1$ and $\lambda_{\mathrm{dice}}=1$ in our experiments, unless otherwise specified.

## 4. Results

### 4.1. Datasets

We applied our method to five distinct retinal vessel segmentation datasets: DRIVE [48], STARE [49], IOSTAR [50], CHASEDB1 [51], and LES-AV [52]. For the training and test set splits of the first four datasets, we adopted the same ratios as LA-Net [53], while for LES-AV, we used the same configuration as RIP-AV [54] for the train–test split. Additionally, we employed the Overlap-tile method for image processing to enhance segmentation performance and increase the number of training samples. Specifically, we divided the original images into smaller patches of size 256×256 with a stride of 128 pixels, resulting in overlapping regions between adjacent patches. This overlap not only helps reduce segmentation boundary errors but also improves the model’s ability to learn fine image details, thereby enhancing segmentation accuracy. A detailed description of each dataset is provided in Table 1.

### 4.2. Implementation Details

We implemented the proposed HM-Mamba using PyTorch version 3.10.9 and conducted all experiments on a GeForce RTX 4090 GPU (NVIDIA Corporation, Santa Clara, CA, USA). Model parameters were optimized using the Adam optimizer with an initial learning rate of $2\times 10^{-4}$. An exponential learning rate decay strategy with a decay factor of 0.98 was employed. To enhance the diversity and effectiveness of training samples, we applied a uniform sliding window strategy across all datasets. Each patch was extracted with a fixed size of 256×256 and a stride of 128, ensuring sufficient contextual overlap between adjacent regions and alleviating discontinuities at patch boundaries. To improve the robustness and generalization capability of the model, we incorporated a series of data augmentation techniques, including color perturbations, random horizontal and vertical flips, and random rotations selected from $\left\{0^{\circ },90^{\circ },180^{\circ },270^{\circ }\right\}$. All augmentation operations were applied consistently to both the input images and their corresponding vessel masks, simulating various imaging conditions and vessel orientations. This strategy effectively mitigates overfitting and enhances the model’s performance on diverse retinal images.

### 4.3. Evaluation Metrics

We evaluated the performance by comparing the predicted segmentation results with the corresponding ground truth labels. The area under the receiver operating characteristic curve (AUC) was calculated, and accuracy (Acc), sensitivity (Sen), specificity (Spe), F1-score (F1), and intersection over union (IoU) were assessed on the binary segmentation maps obtained through thresholding. Their definitions are as follows:

(45) $$\mathrm{Acc}=\frac{TP+TN}{TP+TN+FP+FN}$$

(46) $$\mathrm{Sen}=\frac{TP}{TP+FN}$$

(47) $$\mathrm{Spe}=\frac{TN}{TN+FP}$$

(48) $$F1=\frac{2TP}{2TP+FP+FN}$$

(49) $$\mathrm{IoU}=\frac{TP}{TP+FP+FN}$$

where true positives (TP) and true negatives (TN) represent the number of correctly segmented vascular and non-vascular pixels, respectively, whereas false positives (FP) and false negatives (FN) denote the number of incorrectly segmented vascular and non-vascular pixels.

### 4.4. Comparisons with State-of-the-Art Methods

To thoroughly assess the performance of the proposed method, we conducted a comparative evaluation against ten state-of-the-art (SOTA) approaches: U-Net [55], UNet++ [56], FR-UNet [57], OCT2Former [58], IMFF-Net [59], VM-UNet [37], FRNet [60], OCTAMamba [25], LMFR-Net [61], and U-KAN [62]. To ensure methodological rigor, all experiments followed identical training protocols, including hardware specifications, data augmentation procedures, and optimization schedules, ensuring that any performance discrepancies could be attributed solely to architectural differences.

Table 2 shows the performance of HM-Mamba on the DRIVE dataset, demonstrating superiority over ten state-of-the-art methods. Specifically, HM-Mamba achieves an F1-score of 0.8327, representing a 1.04% improvement over the previous best result by IMFF-Net [59], which had an F1-score of 0.8241. Notably, the IoU metric reaches 0.7164, surpassing IMFF-Net by 2.18%, underscoring significant improvements in segmentation accuracy. Additionally, HM-Mamba attains the highest specificity of 0.9897, surpassing UNet++ [56] and OCT2Former [58] by 0.41%, and it achieves an AUC of 0.9132, exceeding FR-UNet [57] by 0.78%. The overall accuracy also improves to 0.9752, reflecting a 0.55% improvement over UNet++ [56]. These results confirm the robustness of our method in balancing precision and generalization in medical image segmentation tasks.

Table 3 shows the results on the CHASE_DB1 dataset, demonstrating the competitive performance of our proposed HM-Mamba in retinal vessel segmentation tasks. HM-Mamba records the highest F1-score of 0.8197, surpassing the previous best result by IMFF-Net [59] by 1.69%, and achieves an IoU of 0.6839, outperforming IMFF-Net by 1.18%. These improvements highlight its enhanced capability in precise boundary delineation. Additionally, HM-Mamba secures the highest overall accuracy of 0.9760, slightly exceeding UNet++ [56] by 0.02%. While FR-UNet [57] attains a superior sensitivity of 0.8479 and the highest AUC of 0.9154, HM-Mamba delivers competitive performance with a sensitivity of 0.8205 and an AUC of 0.9088. Notably, UNet++ [56] obtains the highest specificity of 0.9880, slightly exceeding HM-Mamba’s specificity of 0.9872. These results collectively demonstrate HM-Mamba’s balanced and robust performance across critical segmentation metrics, underscoring its effectiveness in addressing complex medical imaging challenges.

Table 4 shows the quantitative results on the STARE dataset. HM-Mamba achieves state-of-the-art performance in five out of six evaluation metrics. In particular, HM-Mamba yields a notable improvement in the F1-score, with an increase of 1.34% over FR-UNet [57]. Similarly, it improves IoU by 0.45% and increases sensitivity by 1.77%, both relative to FR-UNet [57]. In addition, HM-Mamba achieves the highest accuracy and AUC, reaching 0.9794 and 0.8995, which surpass the previous best by 0.48% and 0.57%, respectively, highlighting its strong generalization capability. The only exception is specificity, where HM-Mamba records 0.9861, slightly lower than the 0.9898 and 0.9901 obtained by UNet++ [56] and OCT2Former [58]. This indicates potential for improvement in specificity while maintaining competitive performance across other key metrics.

Table 5 presents the quantitative comparison results for the IOSTAR dataset. The proposed HM-Mamba model consistently achieves the highest performance across most metrics, notably delivering the best F1-score of 0.8307, IoU of 0.7121, and AUC of 0.9152. Specifically, HM-Mamba improves the F1-score by 0.88% over FR-UNet [57], the previous best method, increasing from 0.8234 to 0.8307. Similarly, it improves IoU by 1.70%, increasing from 0.7002 to 0.7121, and records the second-highest sensitivity of 0.8386, which is 0.56% lower than that of FR-UNet [57]. Additionally, HM-Mamba achieves the highest ACC of 0.9778 and AUC of 0.9152, surpassing previous best results by 0.56% in ACC and 0.22% in AUC, respectively. These improvements underscore the method’s robustness in segmentation tasks. However, HM-Mamba shows a slightly lower specificity of 0.9848, marginally below OCT2Former’s highest value of 0.9881. These performance gains validate the effectiveness of our hierarchical multi-scale Mamba-based architecture in capturing richer contextual representations for retinal vessel segmentation.

Table 6 provides a detailed comparison of the quantitative results for the LES-AV dataset. The proposed HM-Mamba achieves state-of-the-art performance on five out of six evaluation metrics. Notably, HM-Mamba improves the F1-score by 0.17% over FR-UNet [57], the previous best method, rising from 0.8412 to 0.8427. Similarly, it enhances the IoU by 0.27%, rising from 0.7273 to 0.7293, and it achieves the highest sensitivity of 0.8295, surpassing FR-UNet [57] by 2.04%. Furthermore, HM-Mamba achieves the highest ACC of 0.9817 and AUC of 0.9136, surpassing previous best results by 0.04% in ACC and 0.41% in AUC, respectively. These results underscore the method’s superior capability in segmentation tasks. However, HM-Mamba shows a slightly lower specificity of 0.9933, marginally below OCTAMamba’s highest value of 0.9943. This suggests room for further improvement in specificity while maintaining strong performance across other key metrics.

### 4.5. Qualitative Analysis

**Figure 5:**
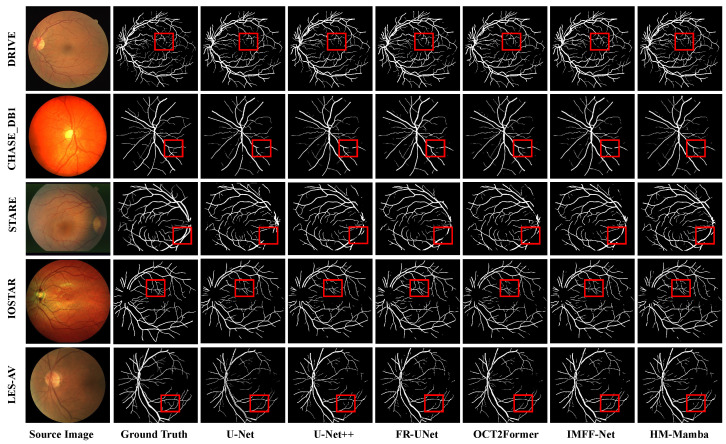
Qualitative comparison of vessel segmentation results across five retinal image datasets. Each row corresponds to a dataset, and each column (from left to right) presents the source image, ground truth, and results produced by U-Net [55], U-Net++ [56], FR-UNet [57], OCT2Former [58], IMFF-Net [59], and the proposed HM-Mamba. Red boxes highlight representative regions containing fine vessels for close-up inspection.

**Figure 6:**
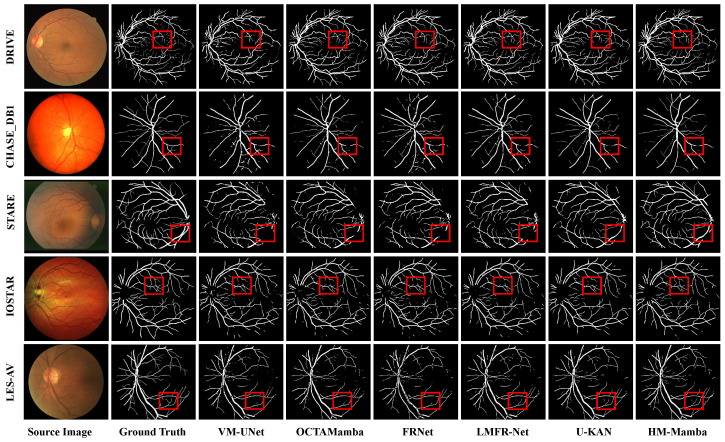
Qualitative comparison of vessel segmentation results on five retinal datasets. Each row corresponds to a dataset, while columns show the source image, ground truth, and results from VM-UNet [37], OCTAMamba [25], FRNet [60], LMFR-Net [61], U-KAN [62], and the proposed HM-Mamba. Red boxes highlight regions containing fine vessels.

Figure 5 and Figure 6 present qualitative comparisons of blood vessel segmentation across five retinal image datasets. Each figure uses datasets as rows, and each column shows the source image, ground truth, and segmentation results from classic algorithms such as U-Net [55], U-Net++ [56], FR-UNet [57], and the proposed HM-Mamba. An intuitive comparison reveals that the proposed HM-Mamba demonstrates notable superiority in performance. Specifically, in fine blood vessel segmentation, HM-Mamba significantly reduces vessel breakage, accurately captures long-range dependencies, and effectively preserves vascular connectivity. Additionally, HM-Mamba exhibits unique advantages in microvascular recognition, effectively identifying more microvessels and fully preserving the spatial structure of retinal vessels. This capability is mainly attributed to the vascular perception convolution module, which captures spatial information, and the multi-scale fusion module in skip connections, which compensates for spatial information loss caused by downsampling, thereby retaining richer microvascular feature details. Overall, across the five datasets, HM-Mamba outperforms competing methods, demonstrating strong performance and promising application potential in retinal vessel segmentation.

**Figure 7:**
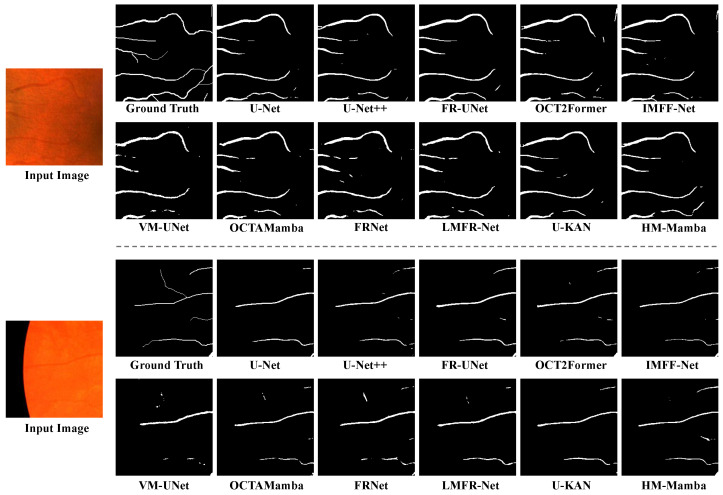
Qualitative evaluation of microvascular visualization results based on patch images.

Figure 7 shows the segmentation visualization results of microvascular areas in retinal images by multiple mainstream and emerging models. Each set of examples includes input images, ground truth, and predicted outputs from 12 models to assess each method’s ability to capture and reconstruct fine vascular structures. Traditional U-Net [55] and its variants exhibit good continuity in extracting major vessels but show noticeable limitations in maintaining the connectivity and integrity of microvessels. Transformer-based architectures (e.g., OCT2Former [58]) outperform convolutional models in preserving details and more accurately reconstruct complex vascular branches. Notably, HM-Mamba, built on the Mamba architecture, demonstrates excellent fine-grained modeling capabilities in both sets of images, particularly in preserving microvascular continuity and integrity, leading to superior structural restoration and stability. In contrast, other models such as U-KAN [62] perform well in recognizing trunk structure but remain somewhat limited in detail recovery. In summary, HM-Mamba delivers the best performance in retinal microvascular segmentation, particularly for medical image analysis applications requiring high structural integrity.

### 4.6. Ablation Study

To comprehensively evaluate the effectiveness of the proposed model, we conducted three ablation studies. These studies aim to quantify the contributions of key modules (MSTS-Conv, MBF-Mamba, HMSI-Mamba), filter parameters (μ, ν), and the dual-branch design within the Multi-branch Fourier-Mamba Block to overall model performance. All experiments were performed on the DRIVE [48] dataset using U-Net [55] as the baseline and evaluated using Dice, IoU, Sen, Spe, ACC, and AUC metrics.

Table 7 illustrates the impact of individual modules and their combinations across different experimental groups. Building on the baseline U-Net [55], introducing individual modules yields measurable gains. For instance, incorporating MSTS-Conv increases the F1 score from 0.8088 to 0.8139 and improves IoU, demonstrating the convolutional module’s effectiveness in enhancing vessel feature extraction and segmentation quality. Furthermore, integrating MBSF-Mamba raises the F1 score to 0.8184 with an IoU of 0.6947, underscoring the Mamba variant’s ability to capture and utilize critical information for this task. When multiple modules are combined, the performance improvement becomes more evident. The combination of MSTS-Conv and MBSF-Mamba achieves an F1-score of 0.8252, surpassing the gains observed with single modules and highlighting their complementary roles in feature representation, thereby enabling a more comprehensive capture of image characteristics. Although adding MSTS-Conv, MBSF-Mamba, and HMSI-Mamba introduces minor fluctuations in certain metrics, the IoU reaches 0.7164 and the AUC attains 0.9132, demonstrating the potential of deep multi-module integration in enhancing overall discriminative power. These results confirm that the proposed modules not only improve performance individually but also significantly enhance segmentation capability on the DRIVE dataset when used in combination.

In our Multi-branch Fourier-Mamba Block, the frequency filters regulate the extraction of low- and high-frequency components through parameters μ and ν, where μ specifies the frequency threshold for the low-pass filter and ν specifies the frequency threshold for the high-pass filter. Table 8 presents the ablation study results on the impact of these frequency filter parameters on retinal vessel segmentation performance. Experimental results indicate that the optimal overall performance is achieved when μ=0.2 and ν=0.8, yielding a Dice score of 0.8327, an IoU of 0.7164, a sensitivity of 0.8318, and a specificity of 0.9897. Consequently, these parameter settings were adopted for the final HM-Mamba model.

Table 9 presents the ablation study results validating the dual-branch design of the Multi-branch Fourier-Mamba Block (MBF-Mamba). Two configurations are compared: (i) Single-branch, where the full input feature $X_{\mathrm{norm}}\in R^{B\times N\times C}$ is processed through two frequency branches (low- and high-frequency), each containing a Mamba layer; and (ii) Dual-branch, which splits the input feature along the channel dimension into two equally sized sub-features $\left(X_{1},X_{2}\right)\in R^{B\times N\times \frac{C}{2}}$, followed by independent Fourier transform, frequency filtering, and Mamba processing before final fusion. As shown in Table 9, the Dual-branch configuration consistently outperforms the Single-branch across all metrics while also reducing the parameter count. The Dual-branch design achieves a Dice score of 0.8327 compared with 0.8275 for Single-branch, an IoU of 0.7164 versus 0.7092, a sensitivity of 0.8318 versus 0.8278, a specificity of 0.9897 versus 0.9855, an accuracy of 0.9752 versus 0.9697, and an AUC of 0.9132 versus 0.9066. Furthermore, we compare the number of parameters of the module with single and dual branches using feature maps of (2, 128, 128, 128), reducing the number of parameters from 0.27M for a single branch to 0.15M for a dual branch. These results indicate that the dual-branch design enhances segmentation accuracy and robustness by effectively leveraging multi-frequency representations while improving parameter efficiency.

### 4.7. Failure Case Analysis

**Figure 8:**
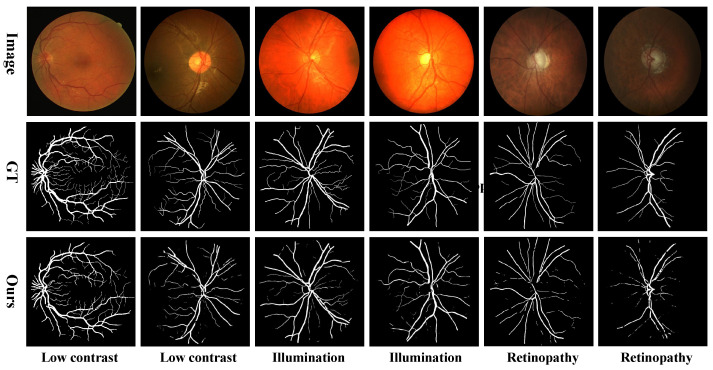
Examples of mis-segmentations under challenging conditions.

Figure 8 illustrates representative failure cases encountered during evaluation. As shown in the third row, our method exhibits a high false-negative rate, often resulting in missing or fragmented vessels. The first four columns illustrate challenges under low contrast and uneven illumination, where subtle vessel-to-background intensity differences and poor lighting reduce vessel visibility, leading to omissions in segmentation. The last two columns highlight the method’s sensitivity to retinal pathologies, where lesions (e.g., exudates, hemorrhages) either occlude vessels or mimic vessel-like patterns, leading to missed or fragmented structures. These cases highlight the inherent difficulty of vessel segmentation in real-world fundus images and underscore the need for more robust feature extraction and context modeling techniques.

### 4.8. Comparison of Parameters, Flops, and Speeds

**Figure 9:**
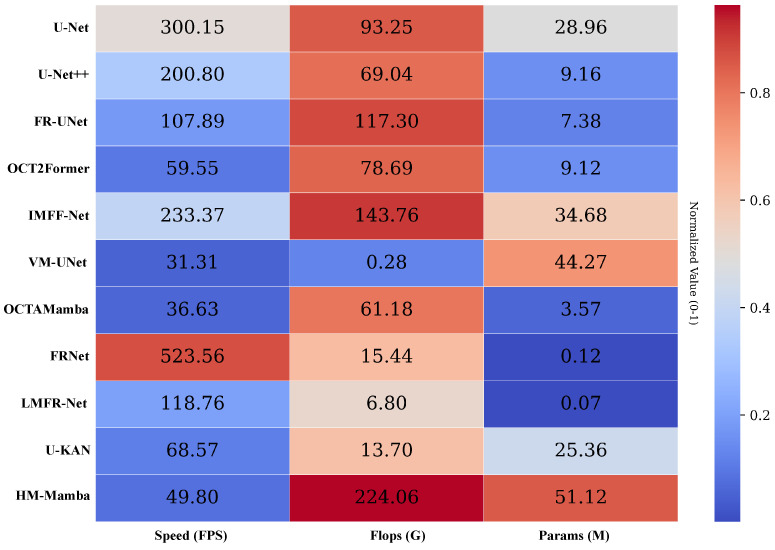
Three aspects of performance for different algorithms: Speed (FPS), Flops (G), and Params (M), with normalized values color-coded for easy visualization. The algorithms compared include U-Net [55], U-Net++ [56], FR-UNet [57], OCT2Former [58], IMFF-Net [59], VM-UNet [37], OCTAMamba [25], FRNet [60], LMFR-Net [61], U-KAN [62], and HM-Mamba.

Figure 9 presents the performance of various algorithms, such as U-Net [55] and U-Net++ [56], across three performance dimensions: speed (FPS), floating-point operations (Flops, G), and parameter count (Params, M). The data are color-coded and normalized for clear and intuitive comparison. In terms of speed, OCTAMamba [25] achieved the highest value, reaching 523.56 FPS, while HM-Mamba runs at 49.80 FPS, which is significantly slower than OCTAMamba but still faster than some algorithms, such as VM-UNet [37]. In terms of floating-point operations, HM-Mamba has a maximum of 224.06 G, which is much higher than most algorithms, such as VM-UNet [37] at only 0.28 G, which indicates that the HM-Mamba operation process is more complex and may have stronger feature extraction capabilities, but it also entails higher computational resource consumption. Regarding the number of parameters, VM-UNet [37] has 44.27 M parameters, while HM-Mamba has 51.12 M, slightly higher than VM-UNet, indicating that the HM-Mamba model is larger in scale. A larger number of parameters can theoretically enable the model to learn richer features, but it also increases training difficulty and storage requirements. Overall, HM-Mamba is slower than some algorithms and has high computational and parameter complexity, resulting in stringent demands on computing and storage resources. In the future, we will explore more efficient parameter optimization strategies to reduce parameter size and resource consumption, thereby enhancing the practicality and general applicability of the algorithm.

## 5. Conclusions

In this work, we propose HM-Mamba, a hierarchical multi-scale network for retinal vessel segmentation. The architecture combines tubular structure-aware convolution with multi-branch Fourier-Mamba modules, enabling the model to effectively capture the elongated continuity of vessels while simultaneously modeling global dependencies and fine-grained features. To further enhance spatial consistency, a hierarchical multi-scale interactive Mamba fusion module is introduced to adaptively integrate multi-level semantic features. Additionally, the model incorporates frequency-domain modulation via dual-branch Mamba units to suppress noise and enhance vessel saliency. Evaluation metrics confirm that HM-Mamba consistently outperforms state-of-the-art methods in both accuracy and structural integrity. While HM-Mamba exhibits strong segmentation capabilities, it incurs higher computational costs due to its multi-stage and frequency-aware design. In future work, we plan to explore lightweight optimization strategies and extend HM-Mamba to 3D vessel segmentation tasks, including magnetic resonance angiography and computed tomography angiography, to further broaden its clinical applicability.

## Figures and Tables

**Table 1:** Description and split of relevant retinal vessel segmentation datasets.

Dataset	Number	Resolution	Train–Test Split	Patches
DRIVE [48]	40	565×584	20–20	320–320
CHASE_DB1 [51]	28	999×960	20–8	980–392
STARE [49]	20	700×605	15–5	300–100
IOSTAR [50]	30	1024×1024	25-5	1225–245
LES-AV [52]	22	1620×1444	11–11	1575–1452

**Table 2:** Comparisons with the state-of-the-art methods on DRIVES dataset.

Method	Date	F1	IoU	Sen	Spe	ACC	AUC
U-Net [55]	MICCAI-2015	0.8088	0.6793	0.7971	0.9838	0.9672	0.8904
UNet++ [56]	MICCAI-2018	0.8230	0.6995	0.8063	0.9857	0.9698	0.8960
FR-UNet [59]	JBHI-2022	0.8233	0.6999	0.8296	0.9826	0.9690	0.9061
OCT2Former [58]	CMPB-2023	0.8199	0.6951	0.8016	0.9857	0.9694	0.8936
IMFF-Net [59]	BSPC-2024	0.8241	0.7011	0.8180	0.9843	0.9696	0.9012
VM-UNet [37]	ArXiv-2024	0.7627	0.6168	0.7245	0.9837	0.9608	0.8541
FRNet [60]	ICASSP-2024	0.8076	0.6778	0.7810	0.9858	0.9677	0.8834
OCTAMamba [25]	ICASSP-2025	0.8165	0.6903	0.7977	0.9854	0.9688	0.8916
LMFR-Net [61]	PAA-2025	0.8194	0.6934	0.8165	0.9835	0.9687	0.9000
U-KAN [62]	AAAI-2025	0.8062	0.6757	0.7889	0.9843	0.9670	0.886
HM-Mamba		**0.8327**	**0.7164**	**0.8318**	**0.9897**	**0.9752**	**0.9132**

*Note:* Bold values denote the best performance across all compared methods.

**Table 3:** Comparisons with state-of-the-art methods on CHASE_DB1 dataset.

Method	Date	F1	IoU	Sen	Spe	ACC	AUC
U-Net [55]	MICCAI-2015	0.8030	0.6713	0.7972	0.9874	0.9753	0.8923
UNet++ [56]	MICCAI-2018	0.8055	0.6751	0.7965	**0.9880**	0.9758	0.8923
FR-UNet [57]	JBHI-2022	0.8052	0.6743	**0.8479**	0.9828	0.9742	**0.9154**
OCT2Former [58]	CMPB-2023	0.8038	0.6725	0.7999	0.9874	0.9754	0.8936
IMFF-Net [59]	BSPC-2024	0.8061	0.6759	0.8180	0.9859	0.9753	0.9020
VM-UNet [37]	ArXiv-2024	0.7386	0.5861	0.7381	0.9829	0.9673	0.8605
FRNet [60]	ICASSP-2024	0.7637	0.6182	0.7616	0.9846	0.9705	0.8731
OCTAMamba [25]	ICASSP-2025	0.7913	0.6552	0.7942	0.9858	0.9736	0.8900
LMFR-Net [61]	PAA-2025	0.7764	0.6351	0.7886	0.9840	0.9715	0.8863
U-KAN [62]	AAAI-2025	0.7993	0.6662	0.8087	0.9857	0.9744	0.8972
HM-Mamba		**0.8197**	**0.6839**	0.8205	0.9872	**0.9760**	0.9088

*Note:* Bold values denote the best performance across all compared methods.

**Table 4:** Comparisons with state-of-the-art methods on STARE dataset.

Method	Date	F1	IoU	Sen	Spe	ACC	AUC
U-Net [55]	MICCAI-2015	0.7755	0.6344	0.7477	0.9873	0.9701	0.8675
UNet++ [56]	MICCAI-2018	0.8023	0.6705	0.7713	0.9898	0.9740	0.8806
FR-UNet [57]	JBHI-2022	0.8130	0.6863	0.8004	0.9885	0.9747	0.8944
OCT2Former [58]	CMPB-2023	0.7980	0.6648	0.7598	0.9901	0.9733	0.8750
IMFF-Net [59]	BSPC-2024	0.8010	0.6694	0.7774	0.9891	0.9736	0.8832
VM-UNet [37]	ArXiv-2024	0.7273	0.5740	0.6534	0.9901	0.9665	0.8217
FRNet [60]	ICASSP-2024	0.7312	0.5820	0.6437	**0.9924**	0.9680	0.8180
OCTAMamba [25]	ICASSP-2025	0.7705	0.6274	0.7194	0.9901	0.9704	0.8547
LMFR-Net [61]	PAA-2025	0.7553	0.6089	0.6820	0.9914	0.9696	0.8367
U-KAN [62]	AAAI-2025	0.7842	0.6456	0.7433	0.9898	0.9717	0.8665
HM-Mamba		**0.8239**	**0.6894**	**0.8146**	0.9861	**0.9794**	**0.8995**

*Note:* Bold values denote the best performance across all compared methods.

**Table 5:** Comparisons with state-of-the-art methods on IOSTAR dataset.

Method	Date	F1	IoU	Sen	Spe	ACC	AUC
U-Net [55]	MICCAI-2015	0.8217	0.6975	0.8050	0.9874	0.9724	0.8962
UNet++ [56]	MICCAI-2018	0.8207	0.6961	0.8061	0.9870	0.9722	0.8966
FR-UNet [57]	JBHI-2022	0.8234	0.7002	**0.8433**	0.9831	0.9715	0.9132
OCT2Former [58]	CMPB-2023	0.8153	0.6883	0.7895	**0.9881**	0.9717	0.8888
IMFF-Net [59]	BSPC-2024	0.8177	0.6919	0.8123	0.9857	0.9714	0.8990
VM-UNet [37]	ArXiv-2024	0.7881	0.6504	0.7684	0.9851	0.9673	0.8767
FRNet [60]	ICASSP-2024	0.8031	0.6712	0.8087	0.9831	0.9687	0.8959
OCTAMamba [25]	ICASSP-2025	0.8122	0.6840	0.8123	0.9846	0.9703	0.8984
LMFR-Net [61]	PAA-2025	0.8077	0.6775	0.7908	0.9863	0.9702	0.8885
U-KAN [62]	AAAI-2025	0.8208	0.6965	0.8107	0.9865	0.9721	0.8986
HM-Mamba		**0.8307**	**0.7121**	0.8386	0.9848	**0.9778**	**0.9152**

*Note:* Bold values denote the best performance across all compared methods.

**Table 6:** Comparisons with state-of-the-art methods on LES-AV dataset.

Method	Date	F1	IoU	Sen	Spe	ACC	AUC
U-Net [55]	MICCAI-2015	0.8353	0.7187	0.7901	0.9936	0.9811	0.8919
UNet++ [56]	MICCAI-2018	0.8388	0.7241	0.8058	0.9940	0.9812	0.9098
FR-UNet [57]	JBHI-2022	0.8412	0.7273	0.8129	0.9918	0.9813	0.9055
OCT2Former [58]	CMPB-2023	0.8355	0.7189	0.7882	0.9938	0.9812	0.8910
IMFF-Net [59]	BSPC-2024	0.8347	0.7175	0.8048	0.9922	0.9807	0.8985
VM-UNet [37]	ArXiv-2024	0.7909	0.6562	0.7399	0.9918	0.9764	0.8659
FRNet [60]	ICASSP-2024	0.7596	0.6172	0.6915	0.9924	0.9741	0.8420
OCTAMamba [25]	ICASSP-2025	0.8154	0.6903	0.7521	**0.9943**	0.9795	0.8732
LMFR-Net [61]	PAA-2025	0.8020	0.6717	0.7574	0.9919	0.9776	0.8746
U-KAN [62]	AAAI-2025	0.8353	0.7182	0.7953	0.9931	0.9810	0.8942
HM-Mamba		**0.8426**	**0.7293**	**0.8295**	0.9933	**0.9817**	**0.9136**

*Note:* Bold values denote the best performance across all compared methods.

**Table 7:** Results of ablation experiments on the DRIVE dataset.

Method	F1	IoU	Sen	Spe	ACC	AUC
Baseline (UNet)	0.8088	0.6793	0.7971	0.9838	0.9672	0.8904
+ MSTS-Conv	0.8139	0.6847	0.8322	0.9782	0.9650	0.9052
+ MBF-Mamba	0.8184	0.6928	0.8225	0.9811	0.9667	0.9018
+ HMSI-Mamba	0.8209	0.6964	0.8312	0.9804	0.9669	0.9058
+ MSTS-Conv	0.8139	0.6847	0.8322	0.9782	0.9650	0.9052
+ MSTS + MBF-Mamba	0.8252	0.7027	0.8264	0.9822	0.9721	0.9093
+ MSTS + MBF-Mamba + HMSI-Mamba	0.8327	0.7164	0.8318	0.9897	0.9752	0.9132

**Table 8:** Ablation study results of frequency filter parameters μ (low-pass cutoff) and ν (high-pass cutoff) on retinal vessel segmentation.

μ	ν	Dice	IoU	Sen	Spe	μ	ν	Dice	IoU	Sen	Spe
0.2	0.2	0.8180	0.6926	0.8153	0.9862	0.6	0.2	0.8218	0.7080	0.8253	0.9856
0.2	0.4	0.8245	0.7076	0.8254	0.9856	0.6	0.4	0.8281	0.7107	0.8264	0.9853
0.2	0.6	0.8209	0.7067	0.8209	0.9860	0.6	0.6	0.8250	0.7068	0.8249	0.9855
0.2	0.8	0.8327	0.7164	0.8318	0.9897	0.6	0.8	0.8277	0.7064	0.8212	0.9859
0.4	0.2	0.8298	0.7151	0.8209	0.9858	0.8	0.2	0.8197	0.7049	0.8209	0.9858
0.4	0.4	0.8242	0.7057	0.8213	0.9858	0.8	0.4	0.8217	0.7078	0.8265	0.9854
0.4	0.6	0.8284	0.7060	0.8237	0.9856	0.8	0.6	0.8239	0.7067	0.8244	0.9855
0.4	0.8	0.8296	0.7063	0.8203	0.9860	0.8	0.8	0.8275	0.7061	0.8234	0.9856

**Table 9:** Ablation study results of dual-branch design in Multi-branch Fourier-Mamba Block.

Input Feature	Dice	IoU	Sen	Spe	ACC	AUC	Params (M)
Single-branch (No Split)	0.8275	0.7092	0.8278	0.9855	0.9697	0.9066	0.27
Dual-branch (Split)	0.8327	0.7164	0.8318	0.9897	0.9752	0.9132	0.15

## Data Availability

The datasets used in this study can be downloaded via the links provided in the referenced papers.

## References

[1] World Health Organization (2019). World Report on Vision.

[2] Qin Q., Chen Y. (2024). A review of retinal vessel segmentation for fundus image analysis. Eng. Appl. Artif. Intell..

[3] Abroug N., Zina S., Khairallah M., Ksiaa I., Kechida M., Ben Amor H., Khochtali S., Khairallah M. (2019). Diagnosing retinal vasculitis and its implications for treatment. Expert Rev. Ophthalmol..

[4] Mitchell P., Liew G., Gopinath B., Wong T.Y. (2018). Age-related macular degeneration. Lancet.

[5] Stolte S., Fang R. (2020). A survey on medical image analysis in diabetic retinopathy. Med. Image Anal..

[6] Rim T.H., Teo A.W.J., Yang H.H.S., Cheung C.Y., Wong T.Y. (2020). Retinal vascular signs and cerebrovascular diseases. J. Neuro-Ophthalmol..

[7] Wu A., Xu Z., Gao M., Buty M., Mollura D.J. Deep vessel tracking: A generalized probabilistic approach via deep learning. Proceedings of the 2016 IEEE 13th International Symposium on Biomedical Imaging (ISBI).

[8] Wu Y., Xia Y., Song Y., Zhang D., Liu D., Zhang C., Cai W. (2019). Vessel-Net: Retinal vessel segmentation under multi-path supervision. Proceedings of the Medical Image Computing and Computer Assisted Intervention–MICCAI 2019: 22nd International Conference.

[9] Wang W., Zhong J., Wu H., Wen Z., Qin J. (2020). Rvseg-net: An efficient feature pyramid cascade network for retinal vessel segmentation. Proceedings of the Medical Image Computing and Computer Assisted Intervention–MICCAI 2020: 23rd International Conference.

[10] Li K., Qi X., Luo Y., Yao Z., Zhou X., Sun M. (2020). Accurate retinal vessel segmentation in color fundus images via fully attention-based networks. IEEE J. Biomed. Health Inform..

[11] Wei J., Zhu G., Fan Z., Liu J., Rong Y., Mo J., Li W., Chen X. (2021). Genetic U-Net: Automatically designed deep networks for retinal vessel segmentation using a genetic algorithm. IEEE Trans. Med. Imaging.

[12] Wei X., Yang K., Bzdok D., Li Y. (2023). Orientation and context entangled network for retinal vessel segmentation. Expert Syst. Appl..

[13] Qin L., Li Y., Lin C. (2025). BINet: Bio-inspired network for retinal vessel segmentation. Biomed. Signal Process. Control.

[14] Shamshad F., Khan S., Zamir S.W., Khan M.H., Hayat M., Khan F.S., Fu H. (2023). Transformers in medical imaging: A survey. Med. Image Anal..

[15] Wang C., Xu R., Xu S., Meng W., Zhang X. (2022). DA-Net: Dual branch transformer and adaptive strip upsampling for retinal vessels segmentation. Proceedings of the International Conference on Medical Image Computing and Computer-Assisted Intervention.

[16] Jiang Y., Liang J., Cheng T., Lin X., Zhang Y., Dong J. (2022). MTPA_Unet: Multi-scale transformer-position attention retinal vessel segmentation network joint transformer and CNN. Sensors.

[17] Li Y., Zhang Y., Liu J.Y., Wang K., Zhang K., Zhang G.S., Liao X.F., Yang G. (2022). Global transformer and dual local attention network via deep-shallow hierarchical feature fusion for retinal vessel segmentation. IEEE Trans. Cybern..

[18] Lin J., Huang X., Zhou H., Wang Y., Zhang Q. (2023). Stimulus-guided adaptive transformer network for retinal blood vessel segmentation in fundus images. Med. Image Anal..

[19] Xu H., Wu Y. (2024). G2ViT: Graph Neural Network-Guided Vision Transformer Enhanced Network for retinal vessel and coronary angiograph segmentation. Neural Netw..

[20] Shi Z., Li Y., Zou H., Zhang X. (2023). Tcu-net: Transformer embedded in convolutional u-shaped network for retinal vessel segmentation. Sensors.

[21] Liu X., Zhang C., Zhang L. (2024). Vision mamba: A comprehensive survey and taxonomy. arXiv.

[22] Wang Z., Zheng J.Q., Zhang Y., Cui G., Li L. (2024). Mamba-unet: Unet-like pure visual mamba for medical image segmentation. arXiv.

[23] Dang T.D.Q., Nguyen H.H., Tiulpin A. (2024). LoG-VMamba: Local-Global Vision Mamba for Medical Image Segmentation. arXiv.

[24] Wang H., Chen Y., Chen W., Xu H., Zhao H., Sheng B., Fu H., Yang G., Zhu L. (2024). Serp-Mamba: Advancing High-Resolution Retinal Vessel Segmentation with Selective State-Space Model. arXiv.

[25] Zou S., Zhang Z., Gao G. (2024). OCTAMamba: A State-Space Model Approach for Precision OCTA Vasculature Segmentation. arXiv.

[26] Cervantes J., Cervantes J., García-Lamont F., Yee-Rendon A., Cabrera J.E., Jalili L.D. (2023). A comprehensive survey on segmentation techniques for retinal vessel segmentation. Neurocomputing.

[27] Zhang H., Fang W., Li J. (2024). A Microvascular Segmentation Network Based on Pyramidal Attention Mechanism. Sensors.

[28] Zhao X., Zhang J., Li Q., Zhao T., Li Y., Wu Z. (2024). Global and local multi-modal feature mutual learning for retinal vessel segmentation. Pattern Recognit..

[29] Tong L., Li T., Zhang Q., Zhang Q., Zhu R., Du W., Hu P. (2024). LiViT-Net: A U-Net-like, lightweight Transformer network for retinal vessel segmentation. Comput. Struct. Biotechnol. J..

[30] Liu H., Yang J., Wang S., Kong H., Chen Q., Zhang H. (2024). Learning to segment complex vessel-like structures with spectral transformer. Expert Syst. Appl..

[31] Gu A., Dao T. Mamba: Linear-Time Sequence Modeling with Selective State Spaces. Proceedings of the First Conference on Language Modeling.

[32] Wang C., Tsepa O., Ma J., Wang B. (2024). Graph-mamba: Towards long-range graph sequence modeling with selective state spaces. arXiv.

[33] Liu Y., Tian Y., Zhao Y., Yu H., Xie L., Wang Y., Ye Q., Jiao J., Liu Y. (2025). Vmamba: Visual state space model. Adv. Neural Inf. Process. Syst..

[34] Ma J., Li F., Wang B. (2024). U-mamba: Enhancing long-range dependency for biomedical image segmentation. arXiv.

[35] Liu J., Yang H., Zhou H.Y., Xi Y., Yu L., Li C., Liang Y., Shi G., Yu Y., Zhang S. (2024). Swin-umamba: Mamba-based unet with imagenet-based pretraining. Proceedings of the International Conference on Medical Image Computing and Computer-Assisted Intervention.

[36] Hao J., He L., Hung K.F. (2024). T-mamba: Frequency-enhanced gated long-range dependency for tooth 3d cbct segmentation. arXiv.

[37] Ruan J., Li J., Xiang S. (2024). Vm-unet: Vision mamba unet for medical image segmentation. arXiv.

[38] Liao W., Zhu Y., Wang X., Pan C., Wang Y., Ma L. (2024). Lightm-unet: Mamba assists in lightweight unet for medical image segmentation. arXiv.

[39] Wu R., Liu Y., Liang P., Chang Q. (2024). Ultralight vm-unet: Parallel vision mamba significantly reduces parameters for skin lesion segmentation. arXiv.

[40] Gong H., Kang L., Wang Y., Wan X., Li H. (2024). nnmamba: 3d biomedical image segmentation, classification and landmark detection with state space model. arXiv.

[41] Jiao L., Wang M., Liu X., Li L., Liu F., Feng Z., Yang S., Hou B. (2024). Multiscale deep learning for detection and recognition: A comprehensive survey. IEEE Trans. Neural Netw. Learn. Syst..

[42] Elizar E., Zulkifley M.A., Muharar R., Zaman M.H.M., Mustaza S.M. (2022). A review on multiscale-deep-learning applications. Sensors.

[43] Srivastava A., Jha D., Chanda S., Pal U., Johansen H.D., Johansen D., Riegler M.A., Ali S., Halvorsen P. (2021). MSRF-Net: A multi-scale residual fusion network for biomedical image segmentation. IEEE J. Biomed. Health Inform..

[44] Tan D., Yao Z., Peng X., Ma H., Dai Y., Su Y., Zhong W. (2023). Multi-level medical image segmentation network based on multi-scale and context information fusion strategy. IEEE Trans. Emerg. Top. Comput. Intell..

[45] Rayed M.E., Islam S.S., Niha S.I., Jim J.R., Kabir M.M., Mridha M. (2024). Deep learning for medical image segmentation: State-of-the-art advancements and challenges. Inform. Med. Unlocked.

[46] Yang D., Liu G., Ren M., Xu B., Wang J. (2020). A multi-scale feature fusion method based on u-net for retinal vessel segmentation. Entropy.

[47] Zhou J., Ma G., He H., Li S., Zhang G. (2025). A multi-scale feature extraction and fusion-based model for retinal vessel segmentation in fundus images. Med. Biol. Eng. Comput..

[48] Staal J., Abràmoff M.D., Niemeijer M., Viergever M.A., Van Ginneken B. (2004). Ridge-based vessel segmentation in color images of the retina. IEEE Trans. Med. Imaging.

[49] Liskowski P., Krawiec K. (2016). Segmenting retinal blood vessels with deep neural networks. IEEE Trans. Med. Imaging.

[50] Zhang J., Dashtbozorg B., Bekkers E., Pluim J.P., Duits R., ter Haar Romeny B.M. (2016). Robust retinal vessel segmentation via locally adaptive derivative frames in orientation scores. IEEE Trans. Med. Imaging.

[51] Owen C.G., Rudnicka A.R., Mullen R., Barman S.A., Monekosso D., Whincup P.H., Ng J., Paterson C. (2009). Measuring retinal vessel tortuosity in 10-year-old children: Validation of the computer-assisted image analysis of the retina (CAIAR) program. Investig. Ophthalmol. Vis. Sci..

[52] Orlando J.I., Barbosa Breda J., Van Keer K., Blaschko M.B., Blanco P.J., Bulant C.A. (2018). Towards a glaucoma risk index based on simulated hemodynamics from fundus images. Proceedings of the Medical Image Computing and Computer Assisted Intervention–MICCAI 2018: 21st International Conference.

[53] Li Y., Zhang Y., Cui W., Lei B., Kuang X., Zhang T. (2022). Dual encoder-based dynamic-channel graph convolutional network with edge enhancement for retinal vessel segmentation. IEEE Trans. Med. Imaging.

[54] Dai W., Yao Y., Kong H., Chen Z.J., Wang S., Bai Q., Sun H., Yang Y., Su J. (2024). RIP-AV: Joint Representative Instance Pre-training with Context Aware Network for Retinal Artery/Vein Segmentation. Proceedings of the International Conference on Medical Image Computing and Computer-Assisted Intervention.

[55] Ronneberger O., Fischer P., Brox T. (2015). U-net: Convolutional networks for biomedical image segmentation. Proceedings of the Medical Image Computing and Computer-Assisted Intervention–MICCAI 2015: 18th International Conference.

[56] Zhou Z., Rahman Siddiquee M.M., Tajbakhsh N., Liang J. (2018). Unet++: A nested u-net architecture for medical image segmentation. Proceedings of the Deep Learning in Medical Image Analysis and Multimodal Learning for Clinical Decision Support: 4th International Workshop, DLMIA 2018, and 8th International Workshop, ML-CDS 2018, Held in Conjunction with MICCAI 2018.

[57] Liu W., Yang H., Tian T., Cao Z., Pan X., Xu W., Jin Y., Gao F. (2022). Full-resolution network and dual-threshold iteration for retinal vessel and coronary angiograph segmentation. IEEE J. Biomed. Health Inform..

[58] Tan X., Chen X., Meng Q., Shi F., Xiang D., Chen Z., Pan L., Zhu W. (2023). OCT2Former: A retinal OCT-angiography vessel segmentation transformer. Comput. Methods Programs Biomed..

[59] Liu M., Wang Y., Wang L., Hu S., Wang X., Ge Q. (2024). IMFF-Net: An integrated multi-scale feature fusion network for accurate retinal vessel segmentation from fundus images. Biomed. Signal Process. Control.

[60] Ning H., Wang C., Chen X., Li S. An accurate and efficient neural network for octa vessel segmentation and a new dataset. Proceedings of the ICASSP 2024—2024 IEEE International Conference on Acoustics, Speech and Signal Processing (ICASSP).

[61] Zhang W., Qu S., Feng Y. (2025). LMFR-Net: Lightweight multi-scale feature refinement network for retinal vessel segmentation. Pattern Anal. Appl..

[62] Li C., Liu X., Li W., Wang C., Liu H., Liu Y., Chen Z., Yuan Y. U-kan makes strong backbone for medical image segmentation and generation. Proceedings of the AAAI Conference on Artificial Intelligence.

